# Guanidine-Based Chloroaluminate Electrolyte: A Candidate for a Rechargeable Aluminum Battery

**DOI:** 10.1021/acs.jpcc.5c02814

**Published:** 2025-07-29

**Authors:** Iwan Sumarlan, Anand Kunverji, Georgina Elliott, Anthony J. Lucio, A. Robert Hillman, Karl S. Ryder

**Affiliations:** † Department of Chemistry, University of Mataram, Jl. Majapahit. No. 62, Mataram 83115, Lombok, Indonesia; ‡ Center for Sustainable Materials Processing, School of Chemistry, 4488 University of Leicester, Leicester LE1 7RH, U.K.; § National Oceanography Center, European Way, Southampton SO14 3ZH, U.K.

## Abstract

Rechargeable aluminum-ion batteries remain an important technological target as an alternative to lithium. Here, acidic room-temperature ionic liquid analogue electrolytes (ILAs) were synthesized from guanidine hydrochloride salt (GuanHCl) and aluminum chloride, with varying metal-to-salt (AlCl_3_/GuanHCl) ratios, and were characterized for their potential application in aluminum-ion batteries. The rheological properties of these electrolytes, including viscosity and electrical conductivity, were determined. The viscosity followed an increasing trend with AlCl_3_ content, while the conductivities followed the inverse trend consistent with Walden’s rule. Both parameters showed an Arrhenius-type behavior with respect to temperature, although, interestingly, the activation energies were all very similar, around 23 kJ mol^–1^. This is comparable to other chloroaluminate liquids and suggests that the mobile charge-carrying species in all the compositions are similar. The speciation of the liquids was investigated by FT-IR and NMR spectroscopies, showing significant trends that indicate interaction between the guanidinium cation and the chloroaluminate center. Electrochemical activities were correlated with rheology, and the 2.0:1.0 formulation exhibited a gravimetric response closest to the Faradaic model. Coin cell testing of the 2.0:1.0 formulation showed interesting trends in cell specific capacity and efficiency as a function of charge/discharge rate that suggests this electrolyte could be a strong candidate for a rechargeable Al battery. The electrolytes are relatively low-cost, making them suitable for potential industrial-scale applications. Clearly, further detailed studies and life-cycle testing of the cells are required in order to realize this technological potential.

## Graphical abstract

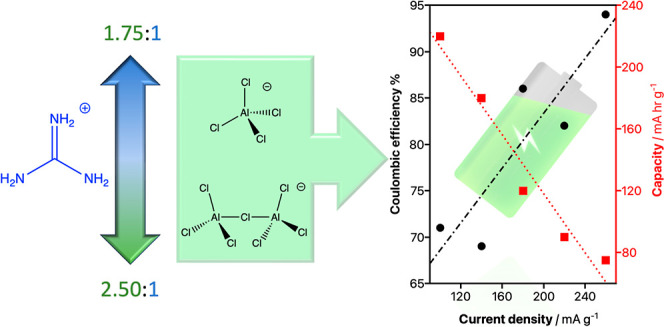

## 1 Introduction

Global energy storage and portable electronic demands are on a steep upward trajectory, driven by consumer demand for mobile devices and the rapid increase in the market for electric vehicles. However, the raw materials for the current state-of-the-art lithium-ion batteries, including Li, Co, and Ni, are becoming increasingly scarce and subject to uncertain market and geopolitical fluctuations.^1^ These factors have sparked research into alternative battery chemistries, causing the emergence of batteries based on Na, K, Ca, Mg, and Al ions. These are abundant, noncritical elements that demonstrate potential as a sustainable, low-cost, efficient alternative to Li-ion batteries to remedy some of the economic issues associated with scarcity and increasing costs of raw materials and to also bolster the circular economy of the battery industry. Aluminum shows promise as an alternative to lithium in many applications; although Al is more than four times heavier than Li, the redox cycle involves 3 electrons, and so, the energy and charge density for the two systems are comparable. Aluminum is highly abundant in the Earth’s crust, it has a very high theoretical volumetric capacity of 8040 mAh cm^–3^, which is four times higher than that of lithium, and has a high gravimetric capacity of 2980 mAh g^–1^.^2,3^ A key area of aluminum ion battery (AIB) development focuses on the challenge of formulating an electrolyte with high Coulombic efficiency and the absence of significant side reactions. The first iteration of an electrolyte utilized in an AIB was water-based, as these hold an inherent safety advantage over nonaqueous systems. Additionally, water-based electrolytes have ionic conductivities approximately 2 orders of magnitude higher than nonaqueous electrolytes (of the type considered here), which is beneficial for the development of high-power systems. Despite these proposed advantages, in practice, utilizing aqueous electrolytes in AIBs incurs fatal problems relating to parasitic, hydrogen-evolving side reactions and the formation of a passivating Al_2_O_3_ layer on the anode, which limits the standard electrode potential of aluminum (−1.662 V vs SHE).^4–6^ As these issues must be resolved in order for AIBs to reach their theoretical promise, recently, many studies have focused on nonaqueous electrolyte development, based on chloroaluminate ionic liquid analogues (ILAs) or deep eutectic solvents (DESs).

The chloroaluminate electrolytes are formed via a Lewis acid–base (LA-LB) reaction, utilizing Lewis acidic AlCl_3_ and a Lewis basic, alkyl, Cl^–^ containing salt.^7,8^ Chloroaluminate melts were the primary ILAs utilized for aluminum electroplating purposes, first employed by Hurley and Wier (1948). The electrolyte was formulated with MCl-AlCl_3_, where M^+^ denotes a monovalent cation, e.g., Li^+^, Na^+^, and K^+^, or an organic cation such as pyrrolidinium or imidazolium. The ionic species present within the ILAs are dependent on the molar ratio of MCl/AlCl_3_; hence, the acidity, basicity, or neutrality of the liquids responds to the LA/LB ratio in solution. In a basic electrolyte, AlCl_4_^–^ and Cl^–^ coexist in equal proportions, and in neutral and acidic melts, the dominant species are AlCl_4_^–^ and Al_2_Cl_7_^–^, respectively.^9,10^ With regard to the reversible Al stripping/deposition process, Al_2_Cl_7_^–^ is the premier species responsible for that reaction. The formation of the dimeric Al_2_Cl_7_^–^ species is correlated with the equilibrium reaction

$$2{AlCl}_{4}^{-}↔{Al}_{2}{Cl}_{7}^{-}+{Cl}^{-}$$

ILAs/DESs demonstrate considerable advantages when compared to aqueous systems, such as low vapor pressure and a wide electrochemical window, which is key in facilitating a highly efficient reversible plating/stripping mechanism, hence making nonaqueous systems more suitable for AIB manufacturing.^11^ Currently, most publications regarding chloroaluminate electrolytes focus on a room-temperature melt of the salt 1-ethyl-3-methylimidazolium chloride (EMIM-Cl) and AlCl_3_, which is commercially available.^12–16^ EMIM-Cl/AlCl_3_ melts exhibit the desired electrochemical and rheological properties; however, the cost of EMIM-Cl makes this electrolyte unfavorable for scale-up. In order to overcome this issue, electrolytes utilizing softer, cheaper Lewis bases, such as urea,^17–20^ acetamidine,^14,21^ triethylamine hydrochloride, pyridinium chloride, and others,^22–24^ have shown promise for scale-up.^14^

In this study, we focus on another low-cost alternative Lewis base in the form of guanidine hydrochloride (Guan). The guanidinium cation is shown below.

Here, we extend our preliminary work comparing guanidine-based liquids to those based on acetamide and urea.^5^ Guanidine is believed to offer potential benefits in chloroaluminate ILAs because the three *N*-atoms of the molecule are structurally equivalent and thought to be a softer Lewis base than oxygen-containing alternatives. This results in a more facile ligand to the aluminum center, improving electrochemical kinetics. In order to develop a more detailed fundamental understanding of these electrolytes, here we present a systematic study of the dependence of the electrochemical performance together with physical and spectroscopic properties on the composition of the liquid. Investigated parameters include varying the LA/LB molar ratios for differing levels of acidity, rheological properties (viscosity and electrical conductivity), speciation determination (NMR and IR), and battery cell performance.

## 2 Experimental Section

### 2.1 Chemicals

The following chemicals were used and sourced accordingly: guanidine hydrochloride (99.99%, Sigma-Aldrich) salt, aluminum chloride (AlCl_3_; anhydrous, granular, 98%, Alfa Aesar), paraffin oil (Puriss, meets analytical specification of Ph. Eur., BP, viscous liquid, Sigma-Aldrich), Carbon Black Super Conductive (Thermo Scientific), graphite (99.99%, Sigma-Aldrich), and PVDF (Rynar HSV 900).

### 2.2 Cathode and Anode Preparation

The cathode was prepared by mixing graphite as an active material, PVD as a binder, and carbon black as an additive in the ratio of 90:5:5 (wt %). All three materials were dissolved in *N*-methyl-2-pyrrolidone (NMP) and then coated on aluminum foil (15 μm thickness) as a current collector. The cathode (diameter 14 mm) was put into the vacuum chamber at 120 °C for 24 h before transferring and storing it in a glovebox. The aluminum-clad anode was cleaned in a deionized water ultrasound bath before vacuum-drying for 24 h at 60 °C.

### 2.3 Electrolyte Synthesis

The electrolytes were prepared on an open bench because synthesis in a sealed glovebox was not practical. In order to reduce exposure of the reactants and liquids to aerobic water, the electrolytes were prepared under a barrier layer of liquid paraffin. This involved the mixing of aluminum trichloride and guanidine hydrochloride at varying ratios of 2.50:1, 2.25:1, 2.00:1, and 1.75:1 (AlCl_3_/Guan) within a liquid paraffin medium over the top. The reaction was initiated with a heat gun until the reaction reached completion. Once the electrolyte formation was apparent, the mixture was further heated at 100 °C on a hot plate for 1 h, following a procedure established in previous studies.^25,26^

### 2.4 ^27^Al NMR and FTIR Investigation

The ^27^Al NMR spectra were acquired using a Bruker AV500 spectrometer at ambient temperature and were corrected for baseline curvature and background noise. A 1.0 M aqueous solution of Al­(NO_3_)_3_·9H_2_O was used as a reference for ^27^Al. The reference solution was placed in a sealed glass insert inside an NMR tube. The FT-IR analysis of the ILAs was conducted using an ATR-IR Bruker Alpha II instrument, controlled by OPUS software.

### 2.5 Simultaneous Cyclic Voltammetry (CV) and Quartz Crystal Microbalance (QCM) Measurements

The CV and electrochemical QCM data were recorded with a Reference600 potentiostat (Gamry) coupled to an eQCM10 M resonator (Gamry), which allowed for the simultaneous acquisition of both voltammetric and acoustic signals. The working electrode was a 10 MHz (±30 kHz) AT-cut quartz crystal resonator (Seiko) with a Pt (electrolyte facing) and Pt (air facing)-coated sides (area 0.2 cm^2^). The Pt surfaces were sputtered to a thickness of 300 nm and polished to a mirror finish, with a surface roughness of approximately 60 nm. The counter electrode was a coiled 2.0 mm diameter aluminum wire (99.9998%, metal basis). The quasi-reference electrode (QRE) was a straight, bare 2.0 mm diameter aluminum wire (99.9998%, metal basis, Alfa Aesar) that provided reproducible potentials against which all potentials were controlled and are reported. The counter and QRE were positioned 1.5 and 1 cm away from the working electrode, respectively. The CV/QCM cell was made in-house to physically fit the resonator electrodes. A PEEK material was used to provide both chemical inertness against the electrolytes examined and robustness for positioning. Electrochemical and QCM data were internally self-consistent and highly reproducible. Adjacent and repetitive CV and EQCM scans were typically overlaid and indistinguishable.

### 2.6 Viscosity and Electrical Conductivity Measurement

The viscosity measurement was conducted in triplicate at 25 °C by means of obtaining the resistance of the electrolyte using a quartz crystal microbalance (QCM 922A) simultaneously during the CV-QCM scan. Using the equation

$$R_{q}=\sqrt{\omega n\rho /2}$$

where ω (=2πf) is the QCM frequency, η is the dynamic viscosity (/g cm^–1^ s^–1^), and ρ is the density (/g cm^–3^), the viscosity of the electrolytes can be obtained.^5,25^ Electrical conductivity was measured via electrochemical impedance spectroscopy (EIS) measurements. The EIS measurements were conducted using a COMPACTSTAT mobile electrochemistry potentiostat (Ivium). Straight, identical-length aluminum wires of 2.0 mm diameter (99.9998%, metal basis, Alfa Aesar) were used for the electrodes. The EIS conductivity cell was calibrated using seven conductivity standards to obtain the cell constant, κ = 0.377 (±0.002) cm^–1^ that was used to convert measured resistance (*R*/Ω) values from ILAs into electrical conductivity (σ/S cm^–1^) values using the equation σ = κ/*R*. Approximately 35 mL of the ILA was pipetted into the glass cell underneath a protective paraffin oil layer. The measurement solution was quiescent. The broadband EIS data were collected at 0 V within a frequency range of 100,000 and 10 Hz with 15 points per decade and an AC voltage amplitude of 10 mV. Complex impedance (Nyquist) data were analyzed by fitting to an electrochemical equivalent circuit consisting of a resistor in series with a constant phase element (i.e., R-CPE). A traditional minimization objective function, a complex nonlinear least-squares method, was used to perform the data fitting.

### 2.7 Linear Sweep Voltammetry (LSV) and Potentiodynamic Polarization

Both LSV and potentiodynamic polarization were conducted using an aluminum wire (99.999%, Alfa Aesar) of 3 mm diameter (area 0.07 cm^2^), sealed in a glass tube with epoxy resin, as the working electrode (WE). Aluminum plates of 2 mm thickness were used as flag-shaped counter electrodes (CE). An aluminum wire (99.999%, Alfa Aesar) was used as the reference electrode (RE).

### 2.8 Battery Preparation

Battery/cell testing was performed with a CR2032 coin cell (Cambridge Energy Ltd.). The constituents of the coin cell included (1) a bottom cap (positive end), (2) cathode (diameter 14 mm), (3) fiberglass paper separator (diameter 16 mm), (4) Al sheet anode material (diameter 16 mm), (5) 0.5 mm stainless steel (SS) spacer, (6) 1 mm SS spacer, (7) SS spring, and (8) SS top cap (negative end). All SS components were of grade 316. The cell was assembled in an argon-filled glovebox with O_2_ and H_2_O < 0.1 ppm.

## 3 Results and Discussion

### 3.1 Viscosity and Electrical Conductivity

The rheological parameters of the viscosity and electrical conductivity play significant roles in battery systems. Both parameters elucidate the electrolytes’ ability to transport mass, hence charge, and help predict the total internal resistance of the system. This can be critical to the performance of a battery cell. Experimentally determined values of viscosity and electrical conductivity, for AlCl_3_-Guan electrolytes of different molar metal/salt ratios are presented in Table 1.

**Table 1:** Viscosity and Electrical Conductivity (Measured at 25 °C) of the Guanidine-Based Chloroaluminate Electrolytes^a^

Al/salt ratio AlCl_3_/GuanHCl	viscosity η/cP	electrical conductivity σ/mS cm^–1^	activation energy *E*_a_/kJ mol^–1^
1.75:1	62 ± 0.1	7.21 ± 0.1	23.0 ± 0.1
2.00:1	70 ± 0.2	6.52 ± 0.1	23.3 ± 0.1
2.25:1	87 ± 0.1	4.74 ± 0.1	25.9 ± 0.1
2.50:1	111 ± 0.1	2.25 ± 0.1	22.8 ± 0.1

a Activation energies were calculated from the slopes of the graphs presented in Figure 1. The experimental errors and uncertainties for these values are defined in detail in the Supporting Information document.

The data show a progressive increase in viscosity as a function of increasing aluminum content. As a consequence, the corresponding decrease in ionic conductivity is observed as the proportion of AlCl_3_ is increased in the melt. These trends are not unexpected because adding AlCl_3_ to the liquid increases the number of metal–salt interactions through charge–charge and covalent mechanisms. At low aluminum concentration, the liquid is Lewis-basic, and the dominant species in such a chloride-rich system is AlCl_4_^–^. As the proportion of aluminum in the melt is increased, the liquid becomes Lewis-acidic and the speciation equilibrium is shifted to favor dimers (Al_2_Cl_7_^–^) and higher oligomers. These higher oligomers are intrinsically less mobile and interact more strongly with the Lewis base salt (here, guanidine).

Furthermore, we have reported earlier^27^ that in an ionic liquid, both the viscosity and electrical conductivity are related to the statistical availability of free-volume “holes” into which mobile species can move. The size of the holes depends on a number of factors including the size and structure of the component ions of the electrolyte, but in general, the greater the concentration of such holes into which charged species can move leads to faster ion migration and more facile ion transport. This results in higher electrical conductivity and lower viscosity. Some insight into this mechanism can be derived from the activation energy for *E*_a_, determined from the ionic conductivity.[Fn fn1] Data reported here agree well with those in a recent study of similar electrolytes over a much narrower range of compositions, where comparable values of viscosity and electrical conductivity were reported for an AlCl_3_/GuanHCl 1.65:1.0 electrolyte of 47 cP and 9.24 mS cm^–1^.^28^

Impedance spectroscopy of the electrolytes was undertaken in the temperature range of 25 °C–80 °C to observe the temperature dependence of electrical conductivity. This investigation allowed us to derive the activation energy from the Arrhenius-type behavior seen by the liquids. The Arrhenius equation can be expressed as

$$\ln\sigma =\ln\sigma_{0}-\frac{E_{a}}{RT}$$

where σ is the conductivity, σ_o_ is a constant, *E*_a_ is the conductivity activation energy, *R* is the general gas constant, and *T* is the temperature in Kelvin.

The temperature-dependent electrical conductivity data for the liquids spanning the range of metal/salt compositions are presented in Figure 1a. The corresponding Arrhenius plots are presented separately in Figure 1b. The increase in temperature, as expected, causes the electrical conductivity of the electrolytes to increase. By increasing the temperature, the charge-carrying species within the electrolyte gains more thermal energy, increasing the statistical availability and free volume of the holes. As the hole mobility of the electrolyte governs charge transport, the higher temperatures enable ions to move into voids of appropriate sizes more easily, resulting in a higher electrical conductivity.^26,29,30^

**Figure 1:**
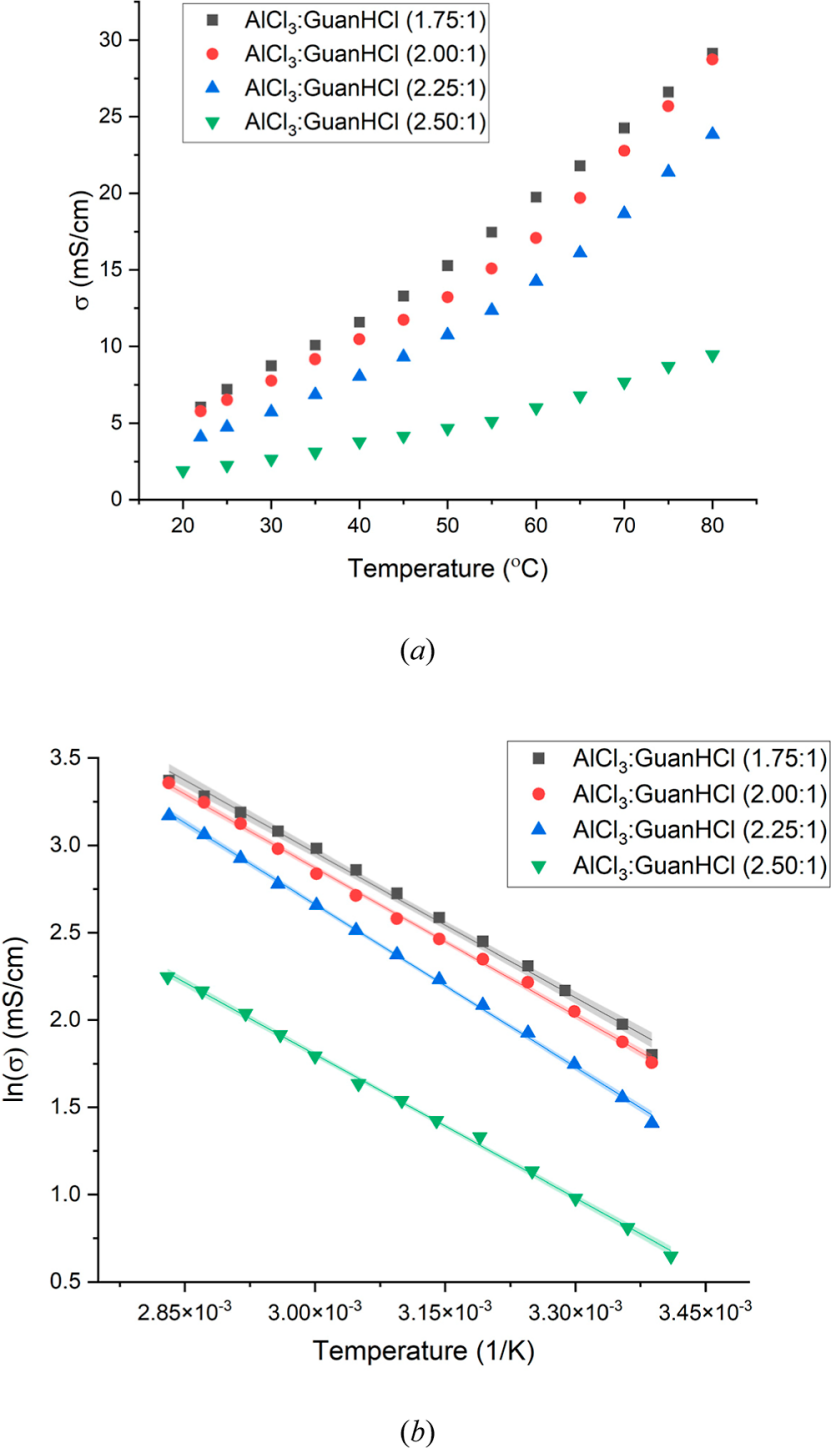
Temperature-dependent conductivities of the different electrolyte formulations; (a) native electrical conductivity data and (b) the corresponding Arrhenius plots from which the conductivity activation energies are calculated (Table 1). The confidence band is indicated by the thickness of the linear correlation for each data set, and the experimental errors and uncertainties for these values are defined in detail in the Supporting Information document.

The data presented in Figure 1 show the anticipated temperature trends, but interestingly, the slopes of the Arrhenius plots, Figure 1b, are very similar for all compositions. The numerical values of the activation energies calculated from these plots are listed in Table 1. The experimentally determined values of *E*_a_ for the different electrolyte compositions are very similar, ranging from 22.8 kJ mol^–1^ to 25.9 kJ mol^–1^. These values lie within the range of approximately 15–30 kJ mol^–1^ for which activation energies for similar systems have been reported, where the value can vary depending on the method of measurement.^5,26^ Hence, the variance observed here with composition probably lies within experimental reproducibility. Recently, a value of 15 kJ mol^–1^ was reported as the activation energy for the diffusion of Al_2_Cl_7_^–^ in an EMIIM-Cl electrolyte.^31^ This may suggest that the AL_2_Cl_7_^–^ ion plays a key role in the behavior of all of these electrolytes.

Additionally, representative data sets of thermophysical data are available in an electronic Supporting Information file accompanying this manuscript.

### 3.2 Composition and Speciation

Speciation analysis of the electrolytes was conducted by using an FT-IR spectrometer with a diamond crystal ATR attachment. The spectra for the various liquids, together with those of neat guanidine hydrochloride (in paraffin oil) for comparison, are presented in Figure 2a. The observed absorption bands for the four electrolytes exhibit a consistent pattern of characteristic peaks. Various vibrational modes for the component species have been identified and reported in recent literature, and some of these are summarized in Table 2.^32–35^ The spectrum of the native guanidine salt, Figure 2a, shows broad, overlapping bands below 750 cm^–1^ that can be attributed to N–H bending modes. However, the NH_2_^+^ and C=N stretching modes are clearly visible at around 1520 and 1600 cm^–1^, respectively. Similarly, the N–H stretching modes are present in this spectrum as broad peaks in the range of 3100–3400 cm^–1^.

**Figure 2:**
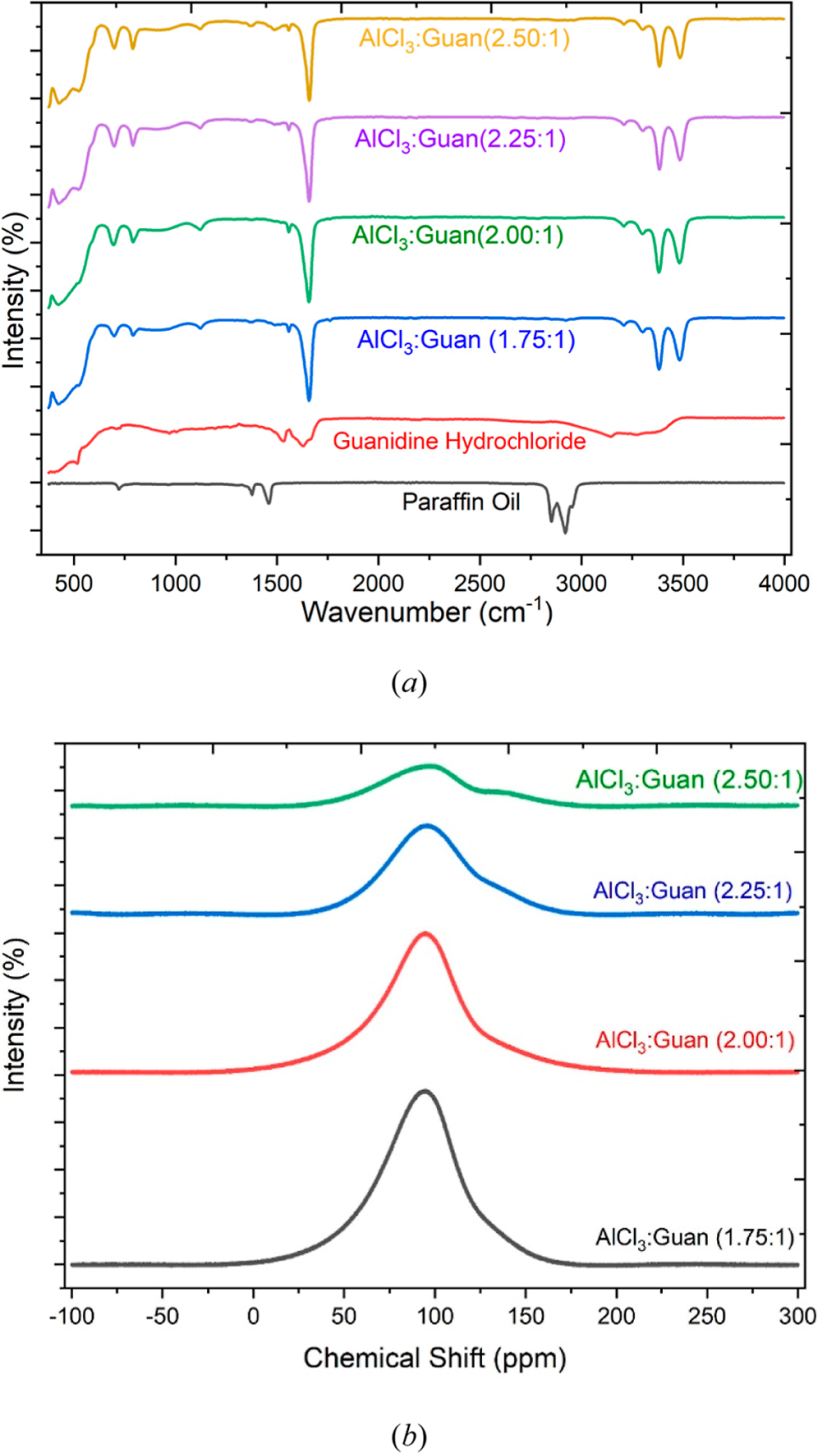
Spectroscopy (r.t., 21 ± 3 °C) of the guanidine chloroaluminate electrolytes of varying composition; (a) FTIR and (b) ^27^Al NMR.

**Table 2:** Vibrational Bands for the Relevant Component Species of the Electrolyte Melts Identified from Various Literature Sources

vibrational mode	wavenumber/cm^–1^	species	reference
N–H bend	710	Guanidinium chloride (in paraffin)	[ ^32–35^ ]
NH_2_^+^ bend	1523		
C=N stretch	1635		
N–H stretch	3146, 3268		
Al–Cl stretch	431, 529	AlCl_4_^–^	[ ^29,36^ ]
Al–Cl stretch	696, 786	AlCl_4_^–^	[^39^
Al–Cl stretch	378, 388, 433	Al_2_Cl_7_^–^	[ ^37,38^ ]

Upon formulation of the electrolyte by mixing with AlCl_3_, new peaks emerge in the spectra, Figure 2a. These are qualitatively similar for the different stoichiometric formulations but show important differences from the native salt. Notably, a broad and intense band spanning the region of 390–620 cm^–1^ was observed. This encompasses the region over which the stretching vibrations of Al–Cl bonds are typically observed (431 cm^–1^ and 529 cm^–1^) and is consistent with the presence of AlCl_4_^–^ species.^29,36^ The aluminum dimer Al_2_Cl_7_^–^ and higher oligomers exhibit absorbance bands between 378–433 cm^–1^,^37,38^ and so, their presence may also be implied, although there are clearly many overlapping bands in this spectral region.

Two additional new peaks were also clearly observed in the spectra of all of the electrolytes at 696 cm^–1^ and 786 cm^–1^, respectively. These can be attributed to stretching modes of the tetrahedrally symmetrical AlCl_4_^–^ ion. Similar vibration modes have been reported for the spectra of [M]­[AlCl_4_] melts (where *M* represents an alkali metal).^39^ Interestingly, these features seem to become more pronounced with increasing AlCl_3_ content (i.e., a higher AlCl_3_/GuanHCl ratio). Another pronounced and interesting feature of all the electrolyte spectra is that the bands corresponding to the C=N stretch (ca. 1600 cm^–1^) and the N–H stretches (ca. 3300–3500 cm^–1^) appear much sharper and better resolved than those in the spectrum of the native salt. This may be a consequence of the disruption of any H-bond interactions in the pure salt and also as a consequence of coordination interaction between the guanidinium cation and the Al-centers.

Additionally, ^27^Al NMR was used to probe speciation of the Al site in the liquids as a function of the metal/salt ratio. Such studies have been widely utilized to analyze the speciation of Al in chloroaluminate ionic liquids and have yielded valuable insights despite the fact that the ^27^Al nucleus (*I* = 5/2) is quadrupolar; this generally results in very broad spectral features. The relatively high viscosity of these liquids limits the isotropic tumbling rates of the molecular species and results in further line broadening. It is widely acknowledged that within chloroaluminate electrolytes, there is an equilibrium between two dominant species shown below.

$$2{AlCl}_{4}^{\text{\_}}↔{Al}_{2}{Cl}_{7}^{-}+{Cl}^{-}$$

The position of this equilibrium as well as the individual reaction rates also affect the appearance of the ^27^Al NMR spectrum. In the case where the salt is also a Lewis base capable of interaction with the Al site, the situation is yet more complex.^5,40^

In our previous study, we have shown the ^27^Al NMR spectrum of the AlCl_3_/GuanHCl 2.0:1.0 liquid in comparison to a series of other chloroaluminate electrolytes.^5^ Here, we show the evolution of the ^27^Al NMR spectrum as a function of metal content, Figure 2b.

The four spectra are dominated by a large, broad feature centered at 105 ppm. As in the other examples cited, the shape of this feature shows broadening originating from a combination of at least two resonance peaks. There is general agreement that in such chloroaluminates, the signal for AlCl_4_^–^ is located at ca. 103 ppm, while the signal for Al_2_Cl_7_^–^ is usually found at lower values, close to 97 ppm. As the ratio of metal/salt is increased, the intensity of this large feature is attenuated. This is partly due to slower tumbling rates of the molecular species resulting from the higher viscosity (Table 1) but also suggests that the Al species in the electrolytes may undergo changes in coordination environments or exhibit a greater diversity of coordination structures as the amount of AlCl_3_ is increased. The broadening of the spectra signifies the presence of multiple Al species with differing local environments, thus highlighting the complexity and dynamic nature of Al coordination in these electrolytes. Additionally, for the most Lewis acidic of the liquids, i.e., 2.25:1.0 and 2.5:1.0 compositions, the central feature looks more divided at around 100 ppm, and additional broad features are present at higher chemical shifts, between 125 and 150 ppm. In our previous reports, we have assigned mixed ligand species such as AlCl_2_L^+^ and AlCl_3_L, where *L* represents an oxygen-donor Lewis base such as urea or acetamide, to ^27^Al NMR lines that appear in the range of 70 – 90 ppm.^5,40^ Here, we speculate that the observed features at a high chemical shift (125–150 ppm) could be adducts of the guanidinium cation with the Al center. In this case, both the formal positive charge and the bidentate[Fn fn2] capability of the nitrogen-based ligand could act as a strongly electron-withdrawing influence on the Al center and shift the ^27^Al resonance to a higher chemical shift.

### 3.3 Aluminum Deposition and Stripping

Guanidine hydrochloride and aluminum chloride were used to formulate the ILA electrolytes, and the ratios tested were 1.75:1, 2.00:1, 2.25:1, and 2.50:1 (AlCl_3_/GuanHCl). During preliminary testing, the ratio 1.50:1 was also tested. However, the electrolyte formed was thermodynamically unstable, forming a brown gel-like substance, and so, it was unsuitable for electrochemical characterization. The cyclic voltammograms of the stable liquid ratios are presented in Figure 3a. The CV studies were combined with QCM microgravimetry and were conducted under controlled conditions at a temperature of 21 ± 1 °C on the platinum-coated face of a 10 MHz AT-cut quartz crystal. In all instances, the cyclic voltammograms exhibited a cathodic current, which is attributed to the reduction of Al^3+^ to Al metal deposited on the surface of the electrode. In the reverse direction, an anodic peak corresponding to the dissolution of Al metal was observed. The presence of a nucleation loop close to the cathodic vertex potential further supported the chemically reversible plating and stripping of Al. These features are consistent with our previous observations on this system.^26^ During repetitive cycling, there were minimal discrepancies observed in the voltammograms of the first and subsequent CV cycles. The *i*(*E*) traces, representing the current as a function of potential, exhibited remarkable linearity during the cathodic current region and the initial segment of the anodic stripping peak. We have previously reported on this phenomenon in other similar systems.^26^ Importantly, no cathodic reduction peak was observed within the potential range depicted in these cyclic voltammograms. Collectively, these findings suggest that the reduction of Al^3+^ and subsequent deposition of Al metal are not restricted by mass transport limitations in any of the tested electrolytes. Across the four examined electrolytes, the onset potential for Al^3+^ reduction was found to be approximately −0.1 V vs Al^3+^/Al. Correspondingly, the electrolytes’ anodic peaks were found to be sharp; however, their magnitudes differed in each ratio. These observations can be attributed in part to the varying concentration of Al^3+^ in the respective liquids, along with the rheological differences, such as electrical conductivity and viscosity.

**Figure 3:**
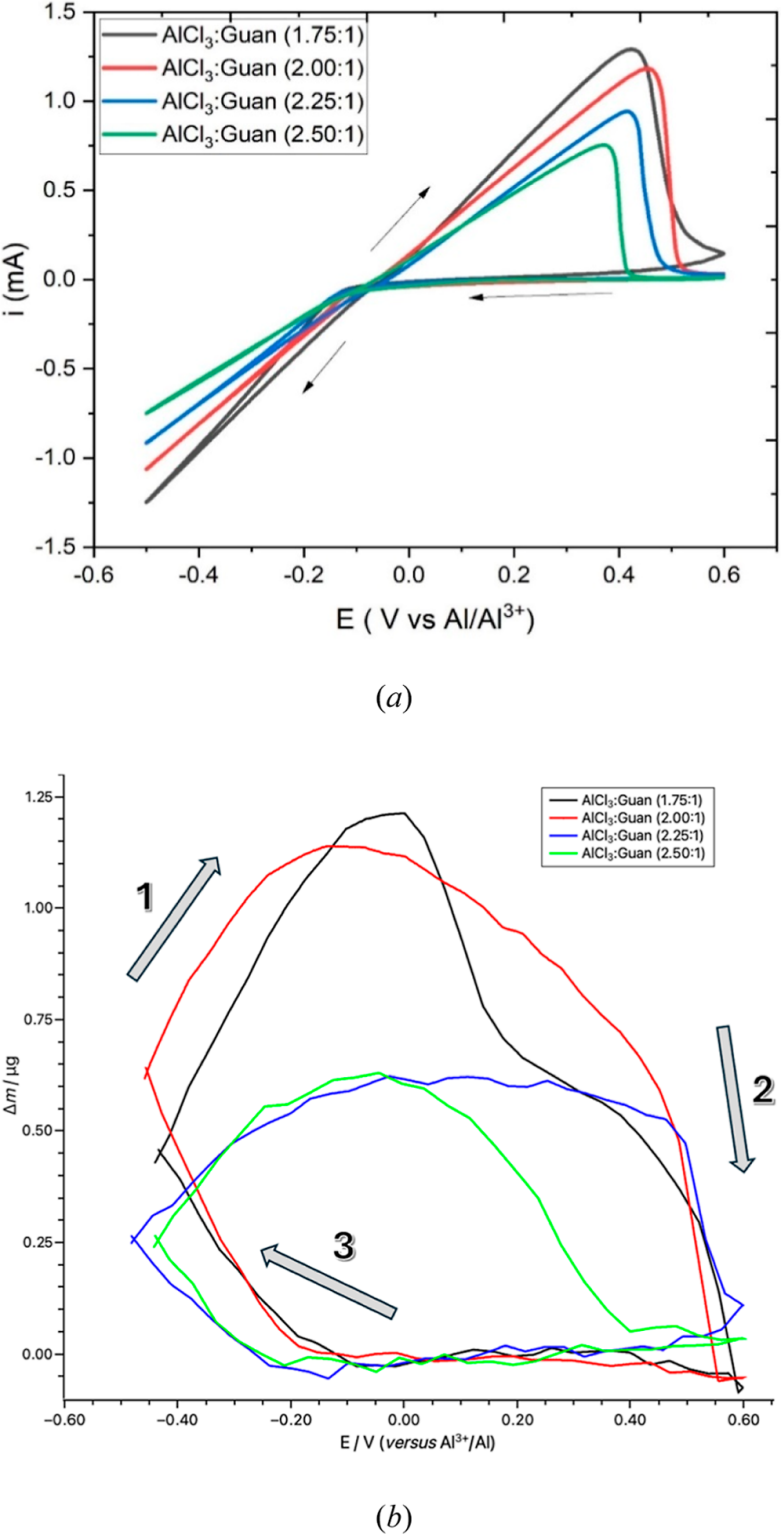
Electrochemical QCM data of the guanidine chloroaluminate electrolytes of varying composition recorded at a temperature of 21 ± 1 °C using a 10 MHz AT-cut quartz crystal with a platinum-coated surface (area 0.2 cm^2^); (a) cyclic voltammetry at a potential scan rate of ν = 10 mV s^–1^ and (b) corresponding mass change (calculated from the Sauerbrey equation) during deposition and stripping observed in the cyclic voltammograms, Figure 3a. The arrows and sequential numbers indicate the direction of the scan.

Within the acidic chloroaluminate environment, the active species responsible for the deposition and stripping of aluminum (Al) are generally attributed to either Al_2_Cl_7_^–^ ^41^ or species such as AlCl_3_L,^42^ where *L* is a Lewis base such as urea, acetamide, or guanidinium. Other important intermediate species are also known to contribute, including AlCl_2_L_n_^+^, where *L* is an uncharged ligand and *n* = 1 or 2. The involvement of these species in the overall mechanism of the reaction can be represented by the reactions below^43^

$$4{Al}_{2}{{Cl}_{7}}^{-}+3e^{-}↔Al+7{{AlCl}_{4}}^{-}$$

Where a Lewis base is also present, this contribution can be illustrated by the following reaction

$$2{AlCl}_{2}{\left(L\right)}_{n}^{+}+3e^{-}↔Al+{AlCl}_{4}^{-}+n\left(L\right)$$

where the Lewis base, *L*, also has a formal charge, as in the case with guanidinium; then, this also contributes to the total charge of the Al complex. In the case of the Lewis-neutral compositions, i.e., a metal/salt of (1:1), limited electrochemical activity is observed. Therefore, it has been suggested that in such liquids, the presence of both Al_2_Cl_7_^–^ and AlCl_2_·(L)_*n*_^+^ is required. The proposed reaction can be visualized as follows

$${AlCl}_{2}{\left(L\right)}_{n}^{+}+2{Al}_{2}{Cl}_{7}^{-}+3e^{-}↔Al+4{AlCl}_{4}^{-}+n\left(L\right)$$

Here, we show that all four electrolytes demonstrated reproducible performance under similar conditions of time (potential scan rate) and temperature, as evidenced by their facile anodic and cathodic responses.

Additionally, the corresponding deposition and dissolution of the aluminum in the electrolytes can be quantified by the frequency change of the QCM crystal using the Sauerbrey relation.^44,45^ According to the Sauerbrey equation, the frequency change (Δ*f*) is directly proportional to the mass change (Δ*m*) on the quartz crystal surface. Representative electrochemical QCM (EQCM) data recorded during cyclic voltammetry are shown in Figure 3b. The four EQCM traces (at a fixed potential scan rate of 10 mV s^–1^) all show rational behavior where the mass increases in the cathodic regime, as Al metal is electrodeposited, and mass is lost during the anodic phase as deposited mass is subsequently electrochemically oxidized and dissolves. The magnitude of mass deposited/dissolved during the cycle is, however, quantitatively different for each liquid. The largest mass change during the single CV cycle (at this scan rate) was observed in the 1.75:1 composition, while the smallest changes were seen in the 2.25:1 and 2.50:1 liquids. These observations correlate with the higher viscosity and lower electrical conductivity of the most Lewis acidic (Al-rich) liquids.

Further quantitative analysis of the EQCM traces can be achieved by integrating the current response from the CV (with respect to time) to yield the total charge as a function of time, *q*(*t*). Subsequent plots of mass versus charge, Δ*m*(*q*), can be compared with the Faraday equation, below

$$\Delta m=\frac{rmm}{zF}\cdot q$$

where Δ*m*/g is the mass change during electrolysis, *rmm*/g mol^–1^ is the relative molar mass of metal deposited, *q*/C is the Faradaic charge, *z* is the electron stoichiometry, and *F*/C mol^–1^ is the Faraday constant. Thus, for a purely Faradaic process, the plot of Δ*m*(*q*) should be linear with a slope of (*rmm*/*zF*). For aluminum, Al^3+^, this has a numerical value of 0.093 μg mC^–1^.

The mass–charge, Δ*m*(*q*), plots for the four electrolytes are presented in Figure 4. These were obtained by combining the integrated *i*(*t*) responses from Figure 3a with the corresponding mass changes shown in Figure 3b. The value of the slopes for these plots is annotated at specific regions on the Δ*m*(*q*) plot, indicated by the dotted linear portions.

**Figure 4:**
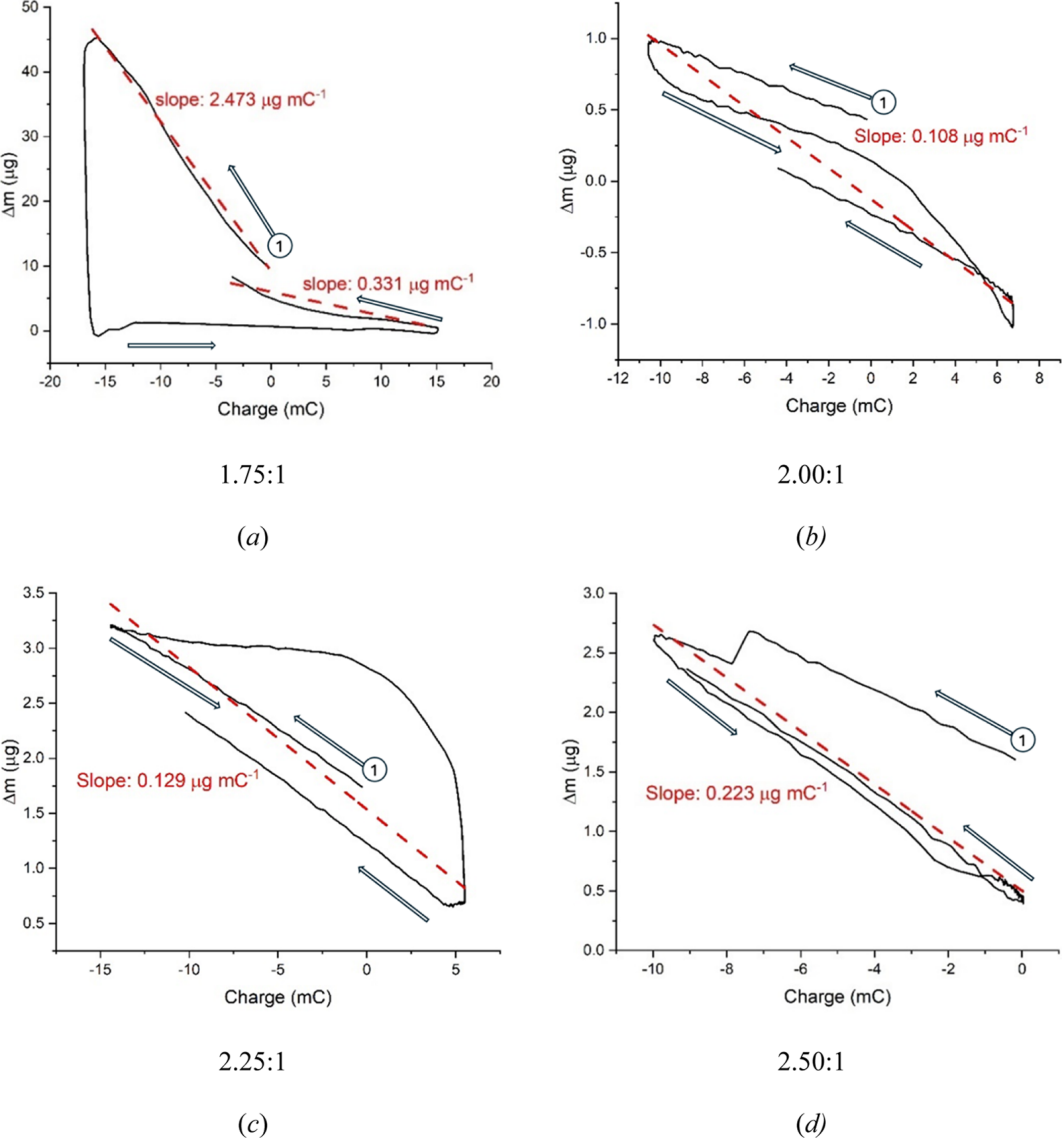
Electrochemical QCM data of the guanidine chloroaluminate electrolytes of varying composition. Mass change, Δ*m*/μg, as a function of charge, *q*/mC, for the electrolytes of different composition from integration of the data presented in Figure 3; (a) 1.75:1, (b) 2.00:1, (c) 2.25:1, and (d) 2.50:1. The value of the slopes for these plots is annotated at specific regions on the Δ*m*(*q*) plot indicated by the dotted linear portions. The start position, ϕ, is shown along with the direction of travel for each scan.

From these traces, we observe that the anticipated linear behavior is not exhibited by any of the electrolytes. In fact, these traces show some complex features, probably indicating that the redox reaction and correlated mass-transfer events are a combination of Faradaic and non-Faradaic processes. The electrolyte that most closely follows the Faradaic trend is the 2.0:1 composition, Figure 4b. Here, the average slope of the Δ*m*(*q*) trace is 0.108 μg mC^–1^, which is quite close to the theoretical value of 0.093 μg mC^–1^. The 1.75:1 and 2.25:1 compositions, Figure 4a,c, respectively, exhibit complex behavior with quite large changes in mass resulting from small changes in charge. A similar effect is sometimes observed during dissolution reactions where mass is separated from a crystal during electrolysis but not electrolytically dissolved—effectively, mechanical detachment. The 2.5:1 electrolyte, Figure 4d, shows quasi-linear behavior, but the measured average slope of 0.223 μg mC^–1^ is more than twice that expected for the Faradaic redox process of Al^3+^. The large positive and negative deviations from the ideal Faradaic linear slope observed here could be due to either dendritic growth, with viscously entrained liquid, or changes in the rheology of the electrolyte as a consequence of the local concentration changes driven by the deposition and dissolution of Al during the cycle. We have already observed that the viscosity and electrical conductivity are quite sensitive to the composition (Table 1). This is an important point that is often overlooked in similar studies. Careful consideration of this effect and the means to mitigate the consequences should feature in future optimization of such electrolytes. It is also possible that other side reactions (minor impurities) could consume charge with no corresponding mass change, but there is no evidence of this in the CVs, as shown in Figure 3a.

Of the four electrolytes examined here, the 2.0:1 composition probably makes the best candidate for a battery electrolyte. The deviations in Faradaic behavior observed could be minimized by extending the time scale of the process, allowing for better equilibration of rheological changes (concentration gradients) during cycling. In practical application, this would mean operating a cell at relatively low current or low power density. This might seem like a compromise in terms of performance relative to current Li-ion technology, but this is not necessarily true because not all application loads demand high power. Other application loads may favor high stability delivered over longer time scales, i.e., low power.

### 3.4 Electrode Passivation

In a practical Al battery cell, Al foil is commonly selected as the cathode/anode material (in charge and discharge, respectively). This is because it is lightweight, inexpensive, and intrinsically compatible with the chloroaluminate electrolyte. However, Al metal is extremely reactive toward oxygen sources and the ingress of water. This can result in passivation of the electrode and, hence, loss of function. To examine this possibility, the linear sweep voltammograms were recorded for the AlCl_3_/GuanHCl electrolyte at an Al foil electrode.

The AlCl_3_/GuanHCl (2.00:1) electrolyte was selected for further detailed investigation because it showed reproducible gravimetric behavior closest to the Faraday model (Figure 3b). Anodic linear sweep voltammetry was employed as a method to explore the potential occurrence of the passivation phenomena on the aluminum electrode. Previous studies demonstrated that passivation can take place in chloroaluminate-based electrolytes, characterized by the solidification of the electrolyte resulting from elevated aluminum concentration near the anode. This phenomenon is primarily attributed to slow diffusion processes. Linear sweep voltammetry of the AlCl_3_/GuHCl (2.00:1) electrolyte is presented in Figure 5a. The potential was scanned from the cathodic limit of 0 V versus the Al^3+^/Al reference, in the positive direction.

**Figure 5:**
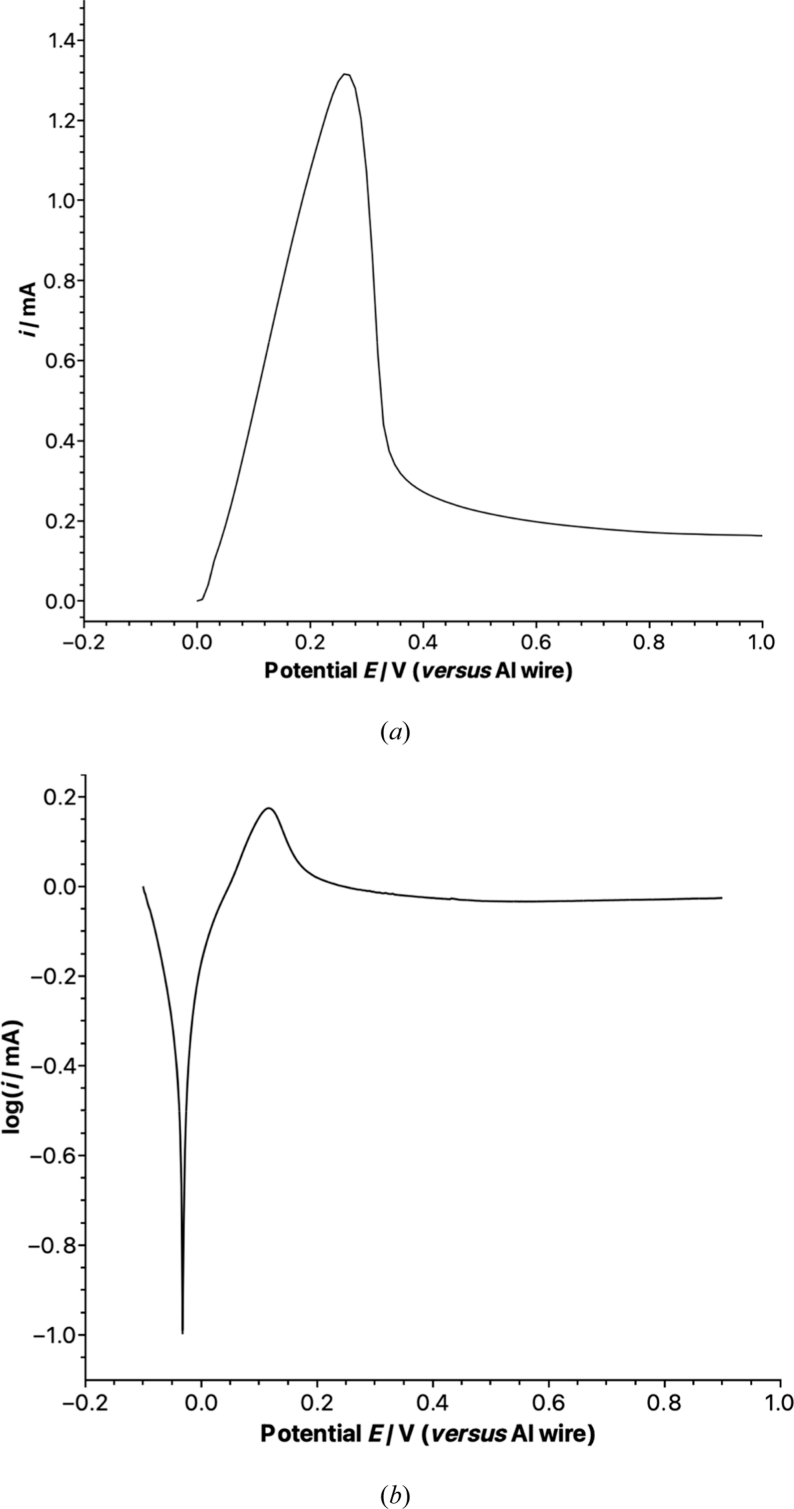
(a) Linear sweep voltammetry and (b) potentiodynamic polarization of the AlCl_3_/GuanHCl (2.00:1.0) electrolyte. Potential scan rate of 10 mV s^–1^. The working electrode was a 3 mm Al disc sealed into the glass (area 0.07 cm^2^), the counter electrode was an Al flag electrode, and an Al bare wire was the quasi-reference electrode.

In Figure 5a, it can be observed that the initial dissolution of aluminum initiates at 0.0 V versus Al^3+^/Al, and the current density experiences a rapid increase as the potential shifts toward more positive values. Once the potential passes +0.13 V, the current density starts to decrease and subsequently levels off to a steady state. The presence of a peak here is evidence of partial passivation of the Al wire electrode. Were this not the case, the dissolution rate would be mass-transfer-dependent and independent of increasing overpotential (see later discussion), giving a current plateau rather than a peak. The magnitude of the steady-state current at potentials > + 0.5 V is evidence that the passivation is only partial. This is consistent with local rheological change (increased viscosity) caused by compositional gradients or with the precipitation of AlCl_3_ on the electrode surface. Consequently, the electrochemical dissolution process of aluminum can be divided into three distinct parts: activation (from 0.00 to 0.13 V), activation–passivation (from 0.13 to 0.27 V), and passivation (beyond 0.27 V). Similar aluminum dissolution processes are also observed in AlCl_3_/urea^46^ and AlCl_3_/EMIM electrolytes.^47,48^

In the activation portion of the dissolution process, the dissolution kinetics of aluminum exhibit an increasing trend in response to the positive shift of potential. The anode dissolution reaction for aluminum in the AlCl_3_-GuanHCl (2.00:1) electrolyte can be represented by the following reaction

$$Al+7{AlCl}_{4}^{-}\to 4{Al}_{2}{Cl}_{7}^{-}+3e^{-}$$

From the equation, it can be concluded that dissolution is favored in electrolytes with higher AlCl_4_^–^ concentration, i.e., Lewis basic melts. In the activation–passivation portion, the dissolution rate of aluminum exhibits a decrease as the potential increases. This phenomenon can be attributed to the formation of a solid AlCl_3_ layer on the surface of the aluminum electrode as the mass-transport layer of the electrolyte is saturated.^48–50^ In the passivation portion (beyond 0.27 V), formation of the passivation layer acts as a diffusion barrier. The presence of this passivation layer plays a significant role in governing the dissolution reaction of aluminum, where the diffusion of AlCl_4_^–^ becomes the primary influencing factor. In this stage of passivation, the limiting current is affected by the position of equilibrium between competing reactions, below^51^

$$\begin{array}{l} Al+7{AlCl}_{4}^{-}\to 4{Al}_{2}{Cl}_{7}^{-}+3e^{-}\\ {Al}_{2}{Cl}_{7}^{-}\to {AlCl}_{4}^{-}+{AlCl}_{3} \end{array}$$

These correspond to dissolution and precipitation processes, respectively.

The potentiodynamic polarization technique is widely used to study corrosion processes, which share many of the mechanistic aspects of electrolytic dissolution. Here, the electrode potential was scanned between two limits (−0.1 V and +0.9 V versus Al wire reference) at a fixed rate, and the logarithm of the magnitude of measured current (or current density) was plotted as a function of potential. The features of such data can indicate the value of the corrosion potential and passivation events. Here, potentiodynamic polarization data are presented in Figure 5b. The plot shows a sharp dip at around −0.02 V that can be associated with the corrosion potential. Additionally, and more significantly here, at +0.12 V, there is a peak and subsequent plateau (at more positive potentials) in the anodic dissolution current, which is strongly indicative of a passivation event. This outcome aligns with similar findings reported previously for EMIM-Cl electrolytes, showing that the passivation behavior can be a strong function of electrolyte composition.^48^ The utilization of potentiodynamic polarization measurements offers valuable insight into the passivation behavior, enabling a comprehensive understanding of the electrochemical processes involved. Moreover, the similarities observed between our results and those previously reported contribute to a growing body of knowledge in this field.

### 3.5 Symmetrical Cell Testing

To evaluate the long-term stability of the interface between aluminum and the electrolyte under dynamic conditions, galvanostatic cycling tests were conducted on symmetrical Al/Al coin cells (where both electrodes were Al foil). A representative voltage profile of a symmetric cell during Al stripping and plating, with a current density of ±0.075 mA cm^–2^ and a time limit of 1 h for charging and discharging, is illustrated in Figure 6.

**Figure 6:**
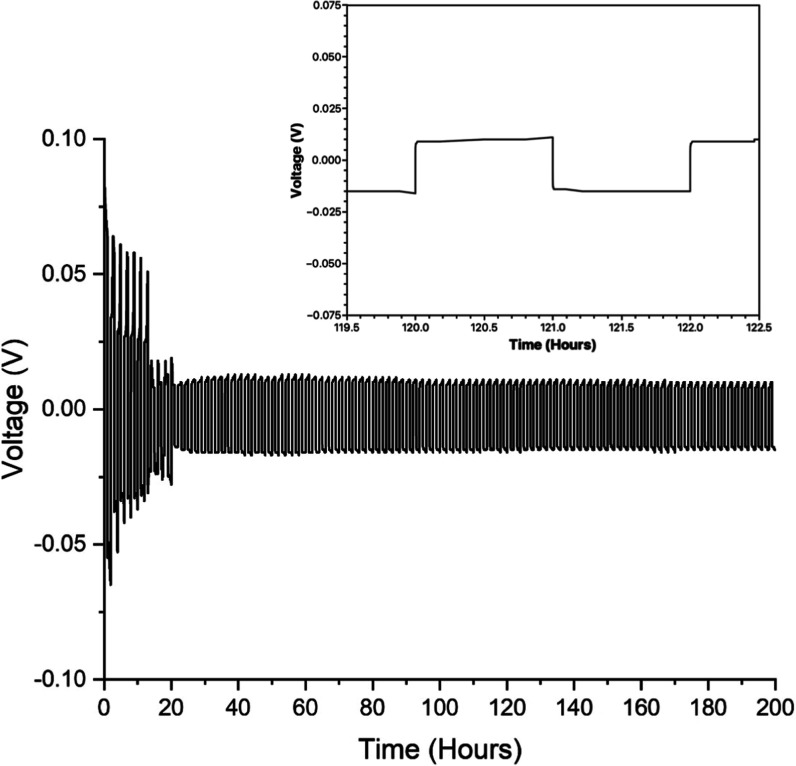
Symmetrical coin cell testing of AlCl_3_/GuHCl (2.00:1). Both the anode and cathode are Al foil. A galvanostatic current density of ±0.075 mA cm^–2^. The inset shows a full galvanostatic cycle in the region where the response has stabilized (120 h < *t* < 122 h).

Upon the application of a negative current through the cell, the voltage profile across electrodes exhibits a flat trend (Figure 6 inset), indicating the release of Al ions from the anode and concomitant electrodeposition on the cathode. Conversely, when a positive current is applied, the voltage profile demonstrates similar behavior in the opposite direction, i.e., the reactions at each electrode are inverted. Initial findings revealed significant overpotentials for symmetric cells, which gradually decreased and reached a stabilized state over time. The observed high overpotential at the initial stage can most likely be attributed to the presence of a passivating layer of aluminum oxide (Al_2_O_3_). Hence, we believe that this native oxide is gradually dissipated during initial cell cycling. During the initial aluminum deposition step, the symmetric cell testing using the electrolyte recorded a maximum overpotential of approximately 75 mV. However, this overpotential gradually decreased to 25 mV after 13 cycles and remained stable at around 10–15 mV after 200 cycles. These results indicate an improvement in the electrochemical performance and the establishment of a more stable interface between Al and the electrolyte over the cycling process, i.e., a conditioning effect. This reduction in overpotential suggests a mitigation of detrimental effects, leading to enhanced stability and efficiency of the symmetrical Al/Al cells during long-term operation. Surface studies of the electrode condition as a function of time would also provide greater insight here and may form the basis of future dissemination.

### 3.6 Full Cell Testing

Finally, the guanidine-based electrolyte was tested in a coin cell configuration. The experimental setup involved the construction of a coin cell, where aluminum foil was utilized as the anode, graphite served as the cathode, and the electrolyte employed was AlC_3_/GuanHCl (2.00:1). The selection of graphite as the cathode material has been successfully employed in the context of aluminum-ion battery applications. Previous studies^52–54^ have demonstrated that graphite can effectively accommodate AlCl_4_^–^ through electrochemical intercalation during the charging process, followed by deintercalation during discharge.^55^ This behavior is similar to that which is currently observed in Li-ion battery technology.

The cells were assembled in an anaerobic glovebox and tested using a range of charge and discharge rates over many cycles, as shown in Figure 7. The data presented in Figure 7a show the cell voltage during charge and discharge as a function of specific capacity/mA h g^–1^. These data were recorded for a range of charge/discharge rates varying from 100 mA g^–1^ to 260 mA g^–1^. The limits of the experimental charging and discharging process for the cells were defined by cutoff voltages, here +2.45 V (charge limit) and +0.25 V (discharge limit), respectively. These data have several important features. The cell voltage (open circuit) of the fully charged cell prior to discharge was consistently close to 2 V. During discharge, the cell voltage decreased, and the rate of decrease depended strongly on the rate of discharge. The slowest discharge rate, 100 mA g^–1^, gave the most stable cell, i.e., the slowest decline in cell voltage. Conversely, the fastest discharge rate, 260 mA g^–1^, showed the fastest decline in the cell voltage. This plot, Figure 7a, also shows the specific capacity achieved for this cell during both charge and discharge. The specific capacity data for this figure are listed in Table 3. Here, it is clear that the highest specific capacities of 310 and 220 mA h g^–1^ (charge and discharge, respectively) were achieved at the slowest charge and discharge rates. Conversely, the lowest specific capacities of 80 and 75 mA h g^–1^ were achieved during the fastest charge and discharge rates.

**Figure 7:**
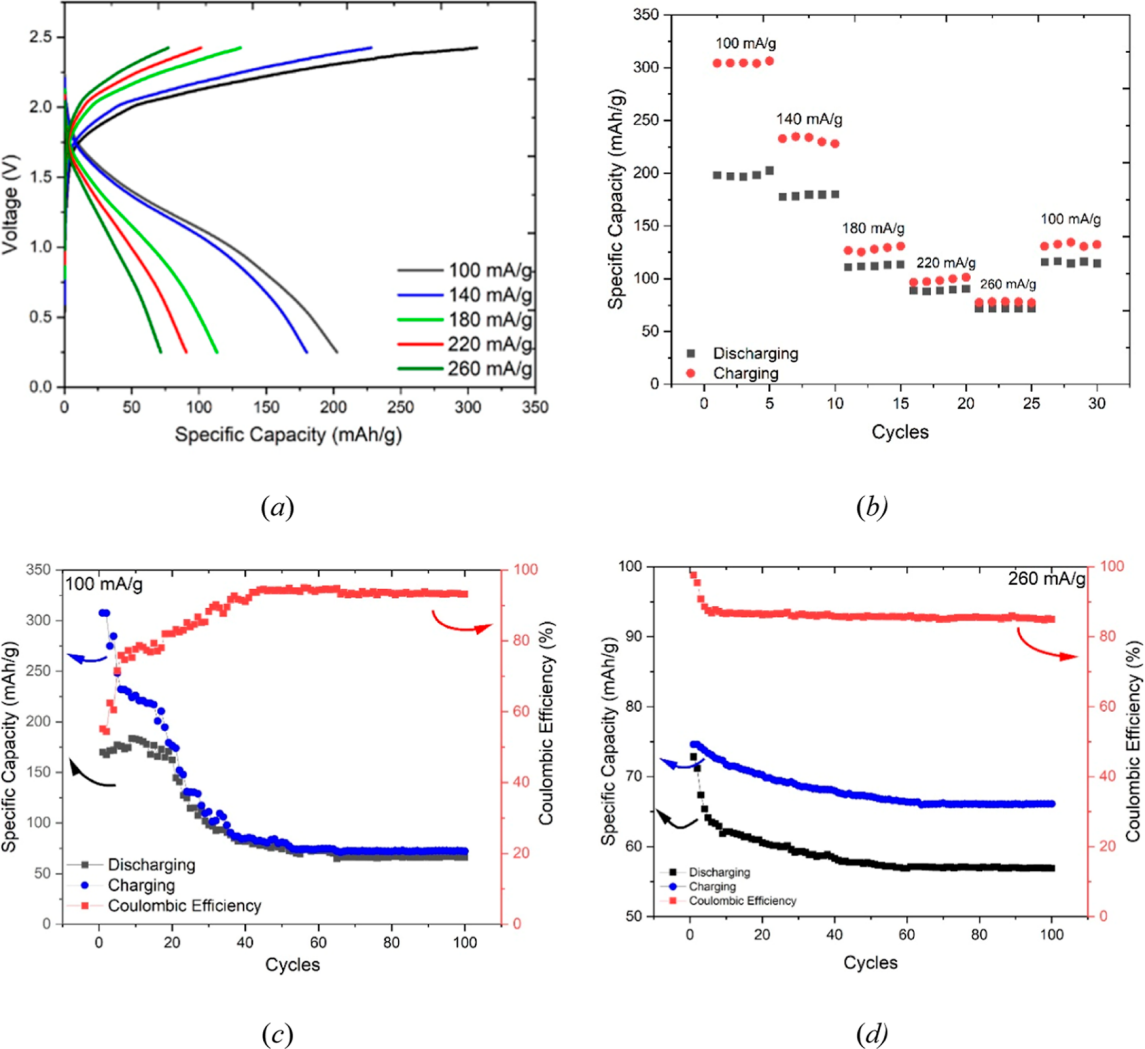
A full coin cell testing profile comprising an Al foil as an anode, spherical graphite as a cathode, and AlCl_3_/GuHCl (2.00:1) as the electrolyte: (a) specific capacity versus cell voltage profiles for a single cell under a range of charge/discharge rates; (b) specific capacity as a function of both the charge/discharge rate and cycle number; (c) specific capacity and Coulombic efficiency as a function of cycle number for a charge/discharge rate of 100 mA g^–1^; (d) specific capacity and Coulombic efficiency as a function of cycle number for a charge/discharge rate of 260 mA g^–1^.

**Table 3:** Specific Capacity and Coulombic Efficiency Data for the Al|(2AlCl_3_/Guan)|Graphite Coin Cell as a Function of the Charge/Discharge Rate^a^

discharge rate/mA g^–1^	specific capacity during charge/mA hr g^–1^	specific capacity during discharge/mA hr g^–1^	Coulombic efficiency
100	310	220	71%
140	260	180	69%
180	140	120	86%
220	110	90	82%
260	80	75	94%

a These data are tabulated from the traves presented in Figure 7a.

The data reported here agree well with those in a recent study of similar electrolytes over a much narrower range of compositions, where comparable coin cell data were reported for an AlCl_3_/GuanHCl 1.8:1.0 electrolyte.^28^ In this study, specific discharge capacities were determined in the range of 100 mA h g^–1^ up to 130 mA h g^–1^ over a range of discharge rates from 100 mA g^–1^ to 5000 mA g^–1^. A similar trend in the dependency of the specific capacity on the rate was observed here, although the highest specific capacity measurement in our study is considerably larger.

This trend is understandable as the combined kinetics of both anion-graphite intercalation and Al deposition/dissolution limit the accessible volume of the cathode and anode, respectively. Consequently, less anode volume and less cathode mass are accessible under faster electron transfer rates. The ratio of charge capacity and discharge capacity represents the Coulombic efficiency of the cell, and these values are also shown in Table 3. Interestingly, the Coulombic efficiency of the cell is quite low, 71% for the slow charge/discharge rate, but increases dramatically, to a maximum of 94%, for the fastest charge–discharge rate.

Considering these observations together, an increase in the discharge rate from 100 to 260 mA g^–1^ reduces the discharge capacity by 66% from 220 to 75 mA h g^–1^. At the same time, the Coulombic efficiency is increased from 71% to 94%. CV of similar liquids over a range of potential scan rates (i.e., current densities) suggests that the Coulombic efficiency of the aluminum redox process is not sensitive to current density. Therefore, we speculate that our observations here are dominated by a cathode (graphite) effect where the most efficient and reversible electron-transfer and mass-transfer processes occur in the outermost interfacial regions of the graphite, which are accessed predominantly at faster discharge rates. Clearly, this warrants further investigation.

In another experiment, the assembled cell was subjected to 5 consecutive charge/discharge cycles at a low current, 100 mA g^–1^. Then the cell was subjected to a further 5 cycles at progressively higher current, up to a maximum of 260 mA g^–1^. The specific capacity data for these experiments are listed in Figure 7b. These show very clearly, as before, that the specific capacity of the cell is reduced with increasing rate but also illustrate the improvement in Coulombic efficiency. For the 5 cycles at 260 mA g^–1^, the charge and discharge capacities are almost coincident, close to 75 mA h g^–1^. In a final 5-cycle series (cycles 26–30, Figure 7b) at the end of this experiment, the cell was again cycled at the lowest rate, 100 mA g^–1^. Here, we observe that the specific capacities do not return to their initial values (cycles 1–5, Figure 7b). This is indicative of capacity fade over the previous 25 cycles and could be due to some irreversible chemical, for example, oxygen ingress, or a physical change in the cell.

To investigate this phenomenon further, a lifetime test comprising 100 cycles was conducted on two separate cells at an applied current of 100 mA g^–1^, Figure 7c, and 260 mA g^–1^, Figure 7d. It can be seen that both the charging and discharging specific capacities decrease until the 52nd cycle, after which they remain stable until the 100th cycle (for the slower 100 mA g^–1^ rate) and until the 61st cycle, maintaining stability until the 100th cycle (for the fastest 260 mA g^–1^ rate). Encouragingly, during the 100 cycles at 100 mA g^–1^, the Coulombic efficiency increases from around 50% to approximately 96%. The initial poor Coulombic efficiency observed in the testing could be attributed to factors such as passivation or the formation of the solid electrolyte interphase (SEI), which can influence the redox reaction and result in a significant gap between the charging and discharging specific capacities. This is perhaps not unexpected because Al foil is manufactured and prepared in air and so will have a thick, dense layer of oxide present initially.

These initial coin cell tests highlight the technological possibilities of the electrolyte here while also giving insight into some of the limiting factors. Clearly, further detailed testing and longer life-cycle analysis are required in order to optimize this technological potential.

## 4 Conclusion

Acidic room-temperature ionic liquid analogue electrolytes (ILAs) with varying ratios of (AlCl_3_/GuanHCl) 1.75:1, 2.00:1, 2.25:1, and 2.50:1 have been successfully synthesized and characterized for their potential application as an electrolyte in aluminum-ion batteries. The rheological properties of these electrolytes, including viscosity and electrical conductivity, were determined. The viscosity followed an increasing trend with AlCl_3_ content, while the conductivities followed the inverse trend, qualitatively consistent with Walden’s rule. The 2.5:1.0 liquid exhibited the highest viscosity of 111 cP and the lowest electrical conductivity of 2.25 mS cm^–1^. Both parameters showed an Arrhenius-type behavior with respect to temperature, although, interestingly, the activation energies (determined from the electrical conductivity data) are all very similar, around 23 kJ mol^–1^. This is comparable to other chloroaluminate liquids and suggests that the dominant mobile charge-carrying species in all the compositions is similar.

The speciation of the liquids was investigated by FT-IR and NMR spectroscopy, showing significant trends that indicate interaction between the guanidinium cation and the chloroaluminate center. These are difficult to attribute to specific speciation changes but do indicate significant shifts in the known speciation equilibria and are consistent with our previous related findings.

The electrochemical analysis of the electrolytes shows that all of the compositions exhibit chemically reversible aluminum reduction with corresponding deposition of Al metal. Electrochemical activity, judged by cyclic voltammetry and microgravimetry, was correlated with rheology, and the 2.0:1.0 formulation exhibited a gravimetric response in comparison to the Faradaic model. Coin cell testing of the 2.0:1.0 formulation showed interesting trends in cell specific capacity and efficiency that are strong functions of the charge/discharge rate. The maximum discharge capacity of 220 mA h g^–1^ was achieved at the slowest rate, while the maximum Coulombic efficiency of 94% was achieved at the fastest charge/discharge rate. These values are comparable with those reported elsewhere.^28^ These trends indicate that mass-transport processes in the cell, such as cathode intercalation and active volume, may limit the capacity and efficiency of the overall reaction. Hence, there remain significant challenges associated with understanding these processes. Additionally, it is also clear that the activity of the Al anode is limited by the native surface oxide and that a consistent response is achieved only after conditioning to remove the passivation layer.

Nevertheless, the combined data here suggest that overall, ionic liquid analogue (ILA) electrolytes based on AlCl_3_ and guanidine hydrochloride show promise as candidates for a rechargeable Al battery. The native rheology and the sensitivity of rheology to compositional changes may limit their use in high-power applications, but the low cost and high abundance of the materials may render them a favorable option for less demanding, low-power applications ubiquitous in portable consumer electronics. These factors also favor scale-up and commercialization. Further detailed studies and life-cycle testing of the cells are required in order to realize this technological potential.

## Supplementary Material



## Footnotes

1 The closely related activation energy for viscous flow can also be obtained from the temperature dependence of viscosity, but this is experimentally much more difficult to measure accurately.

2 The guanidinium cation is trigonal planar with three N atoms, one at each apex of the triangle, but it can only interact with a single Al center through a maximum of two of these; i.e., it cannot be tridentate.

## Notes

Data regarding thermophysical properties of the electrolytes; temperature dependence; linear correlation data error analysis; additional experimental values for electrical impedance; and significant error analysis for computation of viscosity (PDF)
