# Supplementary material for: Guanidine-Based Chloroaluminate Electrolyte: A Candidate for a Rechargeable Aluminum Battery
Source: J Phys Chem C Nanomater Interfaces. 2025 Jul 29;129(31):14154–66. doi: 10.1021/acs.jpcc.5c02814 (PMC12337147; doi:10.1021/acs.jpcc.5c02814)
Supplement: Supplementary file 1 [file jp5c02814_si_001.pdf]

Electronic Supporting Information (ESI)

Guanidine-Based Chloroaluminate Electrolyte: a Candidate for a Rechargeable Aluminium Battery

Iwan Sumarlan [a, b], Anand Kunverji [b], Georgina Elliott [b], Anthony J. Lucio [b, c]
A. Robert Hillman [b] and Karl S. Ryder [b,*]

[a] Department of Chemistry, University of Mataram, Jl. Majapahit. No. 62, Mataram, Lombok, Indonesia.

[b] Center for Sustainable Materials Processing, School of Chemistry, University of Leicester, Leicester, LE1 7RH, U.K.

[c] National Oceanography Center, European Way, Southampton, SO14 3ZH, UK.

[*] Corresponding author

Temperature dependant conductivity data averaged over three separate experiments for each electrolyte formulation together with linear correlation data are presented below.

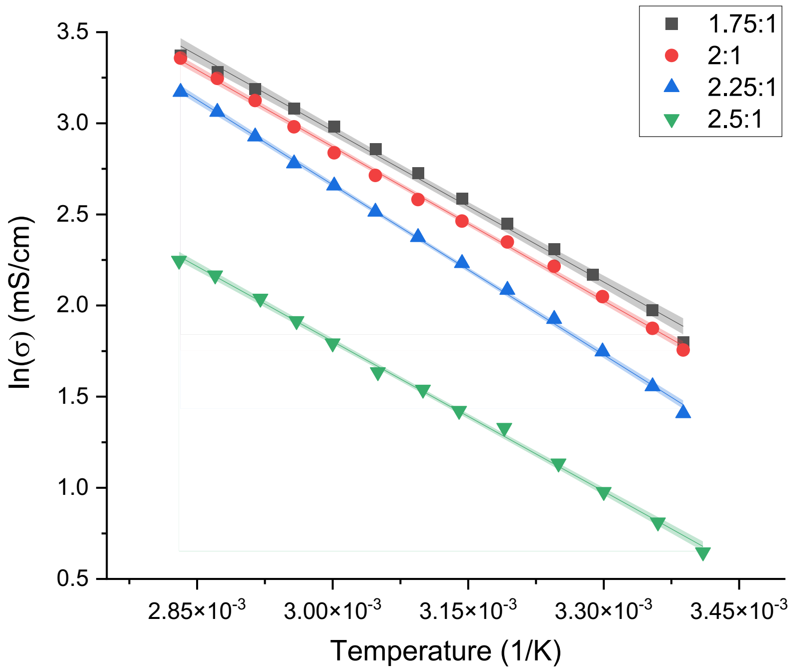

Figure S1. Arrhenius plot for all electrolyte formulations (**Figure 1b**). Each data point was repeated for the runs 3 times and an averaged value from all 3 runs were plotted. Measurements taken from 25-80 °C. Confidence limits and correlations for all data are presented below in **Tables S1** to **S4**.

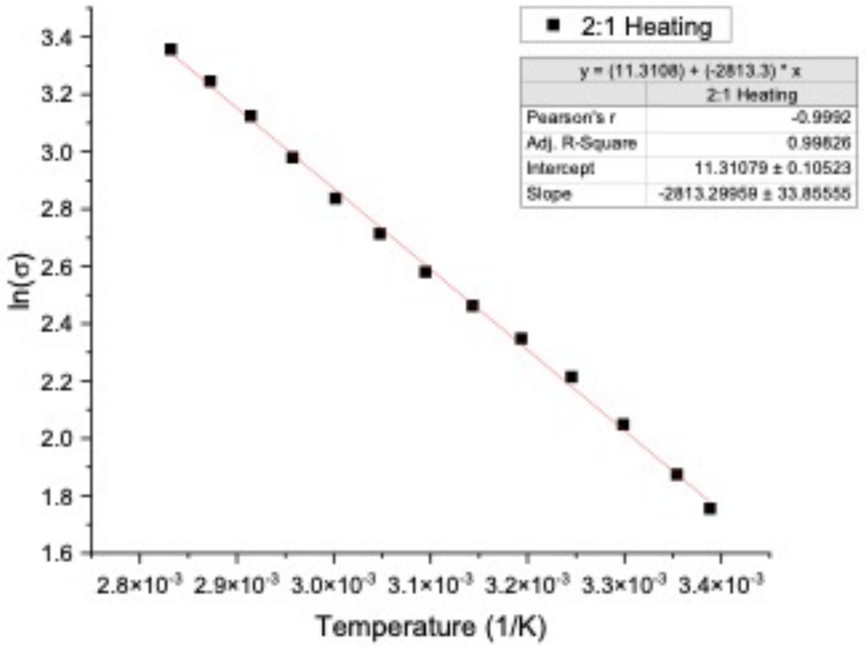

Figure S2. **R**epresentative individual **A**rrhenius plot for 2.0:1.0 electrolyte on heating from 25-80 °C. Calculated E_a is 23.3 kj mol^{-1} as reported. Confidence limits and correlation data are presented in the insert and in **Table S3** below**.**

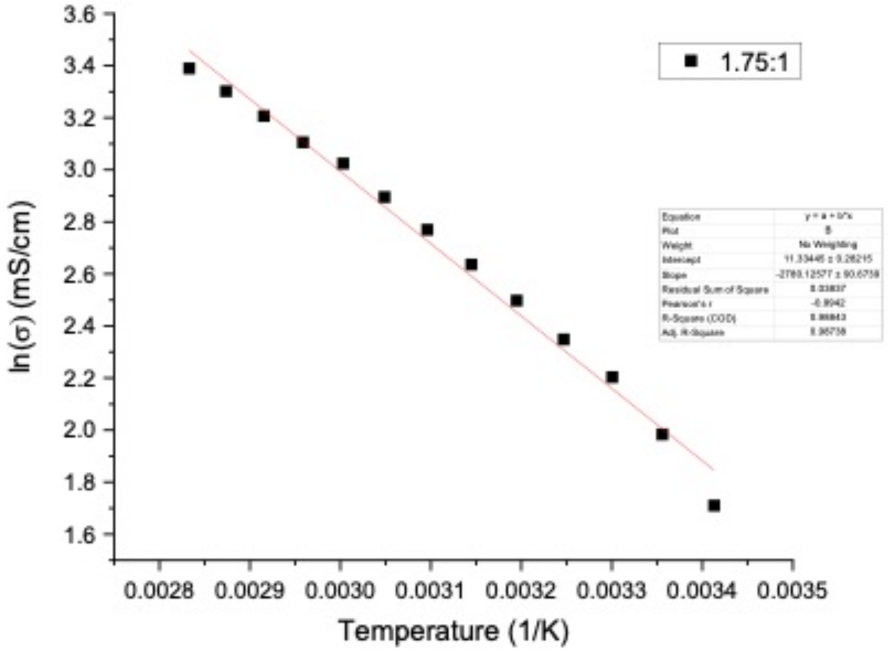

Figure S3. Representative individual Arrhenius plot for 1.75:1.0 on a heating cycle repeat from 25-80 °C. Calculated E_a is 23.1 kj mol^{-1} as reported. Confidence limits and correlation data are presented in the insert and in **Table S4** below**.**

Table S1: Linear correlation data for the 2.5:1.0 electrolyte (**Figure S1**).

**Linear data correlation**			
	**Value**	**Error**	$\mathbf{R^2}$
**Intercept**	10.03504	9.2E-15	1
**Slope**	-2743.86784	2.96E-12	1

**Upper confidence limit**			
	**Value**	**Error**	$\mathbf{R^2}$
**Intercept**	10.04488	0.02194	0.99993
**Slope**	-2740.9443	7.05183	0.99993

**Lower Confidence Limit**			
	**Value**	**Error**	$\mathbf{R^2}$
**Intercept**	10.02519	0.02194	0.99993
**Slope**	-2746.79138	7.05183	0.99993

Table S2: Linear correlation data for the 2.25:1.0 electrolyte (**Figure S1**).

**Linear data correlation**			
	**Value**	**Error**	$\mathbf{R^2}$
**Intercept**	11.98716	9.38E-15	1
**Slope**	-3108.2502	3.017E-12	1

**Upper confidence limit**			
	**Value**	**Error**	$\mathbf{R^2}$
**Intercept**	11.99822	0.0195	0.99996
**Slope**	-3106.34567	6.27348	0.99996

**Lower confidence limit**			
	**Value**	**Error**	$\mathbf{R^2}$
**Intercept**	11.9761	0.0195	0.99996
**Slope**	-3110.15473	6.27348	0.99996

Table S3: Linear correlation data for the 2.0:1.0 electrolyte (**Figure S1 and S2**).

**Linear data correlation**			
	**Value**	**Error**	$\mathbf{R^2}$
Intercept	11.31079	9.37E-15	1
Slope	-2813.29959	3.016E-12	1

**Upper confidence limit**			
	**Value**	**Error**	$\mathbf{R^2}$
Intercept	11.3227	0.021	0.99994
Slope	-2811.24828	6.57693	0.99994

**Lower confidence limit**			
	**Value**	**Error**	$\mathbf{R^2}$
Intercept	11.29888	0.021	0.99994
Slope	-2815.35089	6.57693	0.99994

Table S4: Linear correlation data for the 1.75:1.0 electrolyte (**Figure S1**).

**Linear data correlation**			
	**Value**	**Error**	$\mathbf{R^2}$
Intercept	11.25141	7.62E-15	1
Slope	-2764.26408	2.45E-12	1

**Upper confidence limit**			
	**Value**	**Error**	$\mathbf{R^2}$
Intercept	11.3227	0.03726	0.99979
Slope	-2811.24828	11.9915	0.99979

**Lower confidence limit**			
	**Value**	**Error**	$\mathbf{R^2}$
Intercept	11.29888	0.03726	0.99979
Slope	-2815.35089	11.9915	0.99979

**Al : Salt Ratio** $AlCl_3$:GuanHCl	**Mean impedance** $R_{av.}$ / Ω	**Impedance error** R_{error} / Ω	**Mean viscosity** η / cP	**Viscosity error** η_{error} / cP
1.75:1	1989.3	± 3.0	61.8671	± 0.0932
2.00:1	2116.7	± 5.5	70.0406	± 0.1819
2.25:1	2358.7	± 0.5	86.9717	± 0.0184
2.50:1	2668.0	± 1.0	111.2799	± 0.0417

Table S5: Data for the electrical resistance of the QCM crystal determined for each electrolyte formulation (see experimental Section 2.6). Each measurement of R was carried out three times and presented here are the mean values, $R_{av.}$, together with the error in those measurements, R_{error}. These data were then used to calculate the viscosity values, η, shown, and their associated error, η_{error}. (25 °C)
